# Detailed Genetic Analysis for Identifying QTLs Associated with Drought Tolerance at Seed Germination and Seedling Stages in Barley

**DOI:** 10.3390/plants9111425

**Published:** 2020-10-24

**Authors:** Yasser S. Moursi, Samar G. Thabet, Ahmed Amro, Mona F. A. Dawood, P. Stephen Baenziger, Ahmed Sallam

**Affiliations:** 1 Department of Botany, Faculty of Science, University of Fayoum, Fayoum 63514, Egypt; ysm01@fayoum.edu.eg (Y.S.M.); sgs03@fayoum.edu.eg (S.G.T.); 2 Department of Botany and Microbiology, Faculty of Science, Assiut University, Asyut 71516, Egypt; ahmed.mohamed20@science.aun.edu.eg (A.A.); mo_fa87@aun.edu.eg (M.F.A.D.); 3 Department of Agronomy & Horticulture, University of Nebraska-Lincoln, Lincoln, NE 68588, USA; pbaenziger1@unl.edu; 4 Department of Genetics, Faculty of Agriculture, Assiut University, Asyut 71526, Egypt

**Keywords:** single-marker analysis, barley, drought tolerance index, QTL, PCA

## Abstract

Drought induces several challenges for plant development, growth, and production. These challenges become more severe, in particular, in arid and semiarid countries like Egypt. In terms of production, barley ranks fourth after wheat, maize, and rice. Seed germination and seedling stages are critical stages for plant establishment and growth. In the current study, 60 diverse barley genotypes were tested for drought tolerance using two different treatments: control (0-PEG) and drought (20%-PEG). Twenty-two traits were estimated for seed germination and seedling parameters. All traits were reduced under drought stress, and a significant variation was found among genotypes under control and stress conditions. The broad-sense heritability estimates were very high under both control and drought for all traits. It ranged from 0.63 to 0.97 under the control condition and from 0.89 to 0.97 under drought, respectively. These high heritabilities suggested that genetic improvement of drought tolerance in barley at both stages is feasible. The principal component analysis revealed that root-related parameters account for the largest portion of phenotypic variation in this collection. The single-marker analysis (SMA) resulted in 71 quantitative trait loci (QTLs) distributed across the seven chromosomes of barley. Thirty-three QTLs were detected for root-length-related traits. Many hotspots of QTLs were detected for various traits. Interestingly, some markers controlled many traits in a pleiotropic manner; thus, they can be used to control multiple traits at a time. Some QTLs were constitutive, i.e., they are mapped under control and drought, and targeting these QTLs makes the selection for drought tolerance a single-step process. The results of gene annotation analysis revealed very potential candidate genes that can be targeted to select for drought tolerance.

## 1. Introduction

Water is the essence of life. It is the limiting factor of plant development and crop production, in particular, in arid and semiarid regions. With climate change, drought is a growing constraint for plant growth and crop production. Drought occurs in different regions worldwide, but especially in Africa, Asia, and Australia. Even in Europe, drought is expected to become more severe. Thus, developing drought-tolerant genotypes is an important target for genetic and breeding programs [1]. In terms of production, barley (*Hordeum vulgare* L.) ranks fourth after wheat (*Triticum aestivum* L.), rice (*Oryza sativa* L.), and maize (*Zea mays* L.). In drought-prone regions, cereal seed germination becomes irregular and takes more time [2]. Within cereals, barley is the most abiotic stress-tolerant crop. Therefore, it had been frequently employed in studies to uncover the genetic basis of drought tolerance [3,4]. Seed vigor and early seedling establishment were found to be correlated with higher yields [5]. Drought stress could be lethal to the germinating seeds when the seed moisture level is lower than the critical moisture content [6]. As the plant’s response to drought is very complex, studying drought tolerance is very complex. Drought tolerance during seed germination and seedling establishment in barley are under polygenic control [7,8,9]. The traits related to seed germination and seedling establishment vary markedly under abiotic stress compared to normal growth conditions. Thus, selection for drought tolerance based on a single phenotypic trait would be insufficient [10]. Moreover, uncovering the molecular basis of drought tolerance during seed germination and seedling establishment would allow the development of stress-tolerant genotypes through the disentangling of complex traits.

Compared to the seed germination and seedling establishment stages, a plethora of studies have investigated the genetic control of drought tolerance at late developmental stages in barley. Quantitative trait loci (QTLs) for root-related parameters were identified [11,12,13] for several morphological traits (internode length and leaf length [14,15]) and for physiological parameters (membrane stability [16], osmotic adjustment, proline accumulation, and leaf wilting score [17,18,19,20,21,22], Photosystem II (PSII) activity, gas exchange, relative water content, and electrolyte leakage [23]). As yield is the final target of breeding programs, the effect of drought and the mapping of QTLs for the reproductive stage and seed set have been intensively studied under various conditions [14,24,25,26,27,28,29,30,31,32]. Additional studies have dealt with the effects of drought on flowering time, plant height, and kernel weight [33,34]. Polyethylene glycol (PEG-6000) was frequently used to simulate osmotic stress. PEG-6000 is not absorbed by the plants; thus, the concentration remains constant throughout the experiment, making it the best osmotic stress inducer compared to other osmotic stress inducers such as mannitol and sugar. Moreover, it is a nontoxic osmoticum [35,36,37]. Albeit several studies have investigated the drought effect on seed germination and seedling establishment of barley [38,39,40,41,42,43,44], little attention has been given to identifying QTLs for seed germination and seedling establishment. To the best of our knowledge, few studies have dealt with the identification of the genetic architecture of drought tolerance during seed germination and on seedling vigor [7,8,9]. Thus, the genetic control of drought tolerance during seed germination and seedling establishment is poorly understood. Currently, little is known about the genetic control of seed germination and seedling establishment relative to our knowledge about genetic control of the later stages of barley growth. Therefore, the objectives of the current study are (1) to evaluate the drought tolerance of a diverse set of barley genotypes during seed germination and seedling establishment and (2) to map the QTLs controlling drought stress in these two stages.

## 2. Results

### 2.1. Phenotypic Variation in Germination and Seedling Traits

A set of 60 spring barley genotypes was tested for polyethylene glycol 6000 (PEG)-induced drought tolerance during seed germination and seedling development. Twenty-two phenotypic traits were scored and estimated under both conditions, control (0-PEG) and drought (20%-PEG); the full names, abbreviations, and methods of measurement are listed in Table 1.

**Figure 1:**
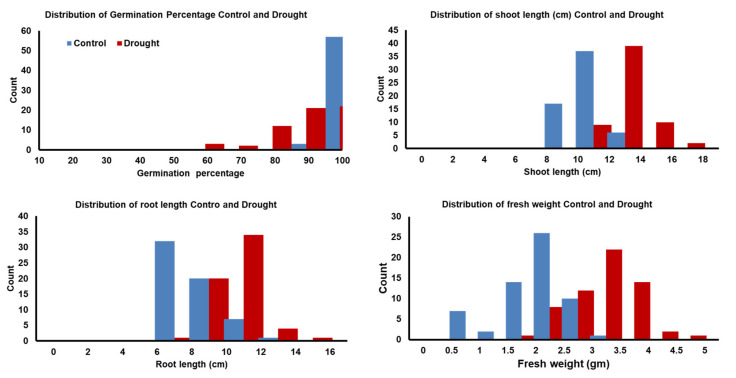
Distribution of traits during seed germination and postgermination development in 60 Egyptian spring barley under control (0-PEG) and drought (20%-PEG).

**Figure 2:**
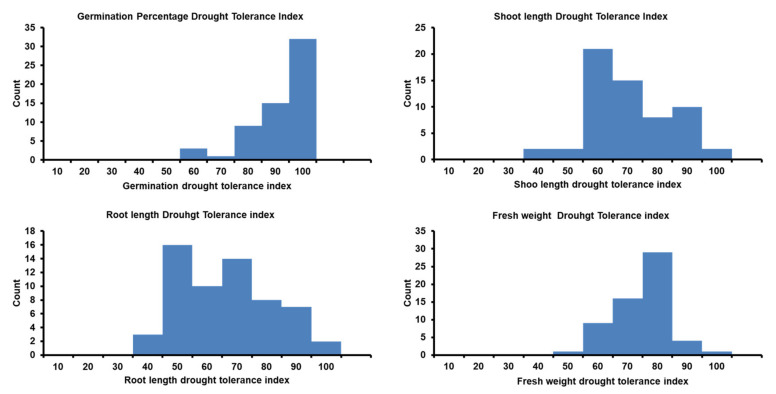
Distribution of drought tolerance index for traits during seed germination and postgermination development in 60 Egyptian spring barley estimated under control (0-PEG) and drought (20%-PEG).

The ranges and mean values of all traits had significant reductions under drought stress compared to their corresponding values under the control treatment, as was expected, except for the shoot–root ratio (SRR; Table 2 and Table S2). Noteworthy, only for germination pace (GP), some genotypes reached the maximum value (GP = 1) after a day. All genotypes had a wide phenotypic variation under both treatments. Germination percentage and GP had higher heritability (H^2^) under drought than under control, while approximate similar heritability estimates were reported in other traits under both conditions (Table S2). Similarly, a wide variation was observed in the calculated parameters among the trait values under control and drought conditions. The distribution of all genotypes for germination percentage (G%), shoot length (SL), root length (RL), and fresh weight (FW) under both conditions is presented in Figure 1. For drought tolerance index (DTI) means, the genotypes’ mean values were 90.20, 83.20, 65.80, 60.90, and 69.50 for G%, GP, SL, RL, and FW, respectively (Table 2, Figure 2). Likewise, the heritability was very high and ranged from 0.88 to 0.95 for GP_DTI and RL_DTI, respectively. For reduction indices, the genotypes’ mean values were 9.72, 0.16, 4.47, 4.30, and 1.11 for G%, GP, SL, RL, and FW, respectively (Table S2). The heritability was very high and varied from 0.83 for Reduction_GP to 0.95 for Reduction_RL (Table S2).

The analysis of variance revealed a highly significant variation (*p* ≤ 0.01) among genotypes for all traits under both treatments (Table 2). To estimate the contribution of each trait to the total observed variance, a PCA analysis was conducted. The eigenvectors resulting from the PCA analysis showed that under both treatments, the RL criteria contributed a large portion of the total variation maintained in this collection (data not shown).

The DTIs for the five traits—G%, GP, SL, RL, and FW—were used to select the most drought-tolerant genotypes at the seedling stage as well as at the adult stage (Table S6). The 10 most drought-tolerant genotypes were selected in each DTI. Then, the genotype was finally selected if it was among the 10 most drought-tolerant genotypes in at least two DTIs. As a result, in the current study, a set of 13 genotypes were assigned as drought-tolerant. Four genotypes, SCSAL-21, PNBYT15, SCCAL-36, PNBYT1, and SC4-41, were found to be among the 10 most drought-tolerant genotypes in three DTIs, while nine others were the most drought-tolerant in two DTIs (Table 3).

### 2.2. Correlation Analysis

**Figure 3:**
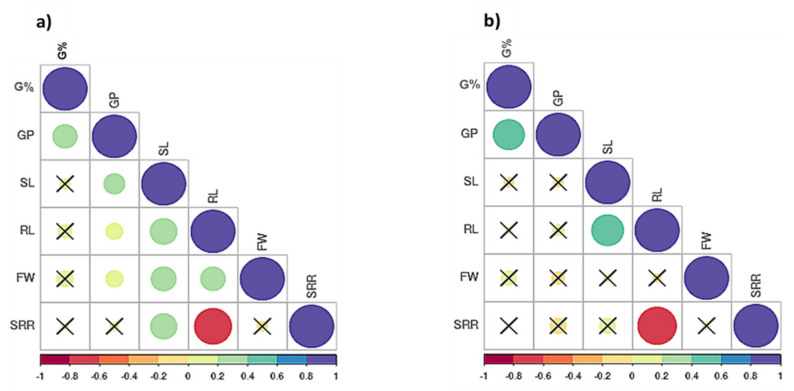
Correlations of the traits in 60 Egyptian spring barley, (**a**) under control and (**b**) drought stress (20% PEG). G% = Germination percentage, GP = Germination pace, RL = Root length, SL = Shoot length, FW = Fresh weight and SRR = Shoot-Root length ration. Where (X) stands for the non-significant correlations.

Pearson’s correlations were computed for all traits using the mean values (Figure 3a,b). Under the control condition, all significant correlations were positive except for the correlation between SRR and RL, with r = −0.70 ** (Figure 3a). Germination percentage (G%) had positive and significant correlations only with GP under both treatments (Figure 3a,b). Under drought stress, a positive and significant correlation was found between SL and RL, with r = 0.52 **. SRR and RL had a negative and significant correlation, with r = −0.75 ** (Figure 3b).

For all traits, the phenotypic correlations between their corresponding DTIs and their reduction indices were negative and significant (Figure S1). Among DTIs, the highest positive and significant correlations were observed between G% and GP, as well as between SL and RL. Similarly, for reduction indices, G% with GP and RL with SL showed the highest positive and significant correlations.

### 2.3. Marker–Trait Association (QTL Analysis)

**Figure 4:**
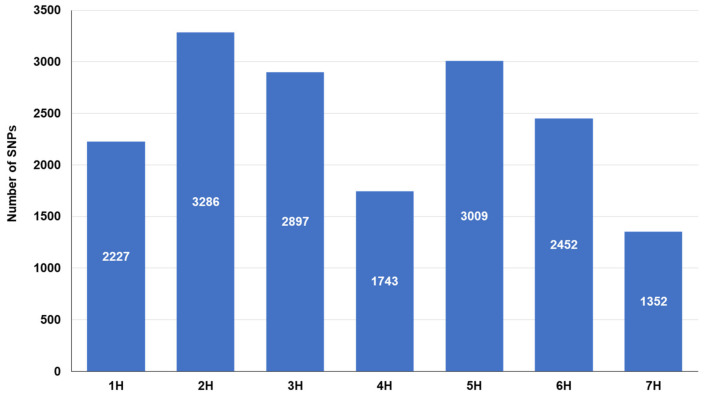
Distribution of SNP markers resulted from GBS across barley chromosomes.

The distribution of the single nucleotide polymorphism (SNP) markers on each chromosome is presented in Figure 4. The SNP markers ranged from 1352 (7H chromosome) to 3286 (2H chromosome).

**Figure 5:**
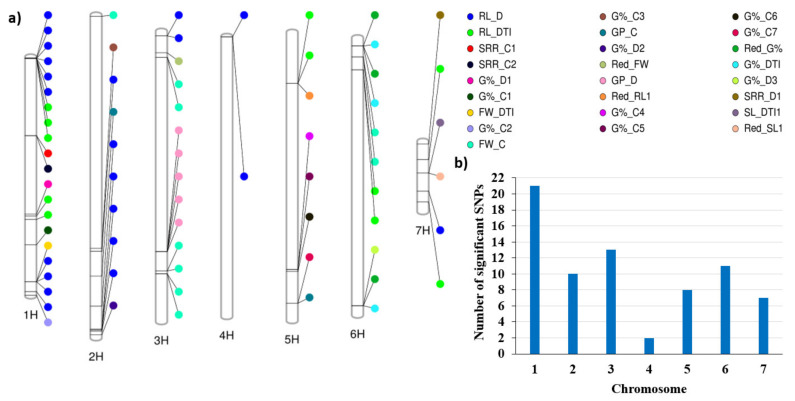
(**a**) the distribution of QTL detected under drought and control conditions across all barley chromosome, (**b**) the number of QTLs on each chromosome.

The single-marker analysis (SMA) resulted in 71 significant marker–trait associations controlling 17 traits (Table S3). The summary of the SMA results is presented in Table 4. No QTLs were identified for shoot length under the control condition (SL_C) or under drought (SL_D), root length control (RL_C), or Red_GP and GP_DTI. The number of QTLs was distributed unevenly across the seven barley chromosomes (Figure 5a,b). Chromosome 1H had the highest number of QTLs (21), while chromosome 4 had only 4 QTLs. Several hotspots of QTLs were identified across all chromosomes except chromosome 4H. For example, for the root-related traits, on chromosome 1H, two hotspots of QTLs were detected. The first included 4 QTLs in the region between 1,828,706 to 2,838,678 bp. The second hotspot enclosed 4 QTLs; this region extended between 436,585,416 to 455,758,766 bp. Additionally, on chromosome 2H, another hotspot for root length under drought included 4 QTLs and extended from 609,969,235 to 614,052,626 bp (Table S3). The number of QTLs detected under drought stress (n = 35) was higher than those detected under control conditions (n = 15). Under drought stress, 21 QTL were found for root length, with R^2^ ranging from 26.9% to 44.3%, while under control conditions, seven QTLs were found to be associated with G%, with R^2^ ranging from 29.1% to 47% (Table 4). Figure 5 shows that chromosomes 1H and 2H can be targeted to select for root length under drought; chromosome 3H is suitable to select for fresh weight under control as well as for germination pace under drought, whilst chromosome 5H harbors QTLs that can be harnessed to improve G%.

#### 2.3.1. QTLs for G% Traits

The single-marker analysis resulted in ten QTLs: seven for G%_C and three for G%_D. The QTLs for control were localized on three chromosomes: 5H (4), 1H (2), and 2H (1). The QTL G%_1H_C2 explained 47% of the phenotypic variation of G%_C. For G%_D, three QTLs were distributed on chromosomes 1H, 2H, and 3H. The QTL G%_1H_D1 accounted for 42% of the phenotypic variation (Table S3). For G%-related indices, three QTLs for G%_DTI and three QTLs for Red_G% were detected. Notably, all of them were mapped on chromosome 6H. The most effective QTLs, G%_6H_DTI3 and Red_6H_G%4, contributed to 33% and 33% of the phenotypic variation for G%_DTI and Red_G%, respectively. Noteworthy, the SNP marker S6_520541285 is attributed to three QTLs controlling G%_D, G%_DTI, and Red_G%. These results suggest that this marker resides in a genomic region with pleiotropic effects and harbors genes controlling the variation of more than one trait under contrasting growth conditions.

#### 2.3.2. QTLs for GP Traits

Seven QTLs were detected: two for GP_C on chromosomes 2H (1) and 5H (1), and five QTLs for GP_D; all of them resided on chromosome 3H. For GP_C, the QTL GP_5H_C2 explains 46% of the phenotypic variation. For GP_D, five QTLs were clustered at a region from 428,008,275 to 428,278,949 bp (Table S3).

#### 2.3.3. QTLs for RL Traits

The highest number of QTLs for a single trait was detected for root length under drought (RL_D) with 20 QTLs. The QTLs for RL_D were distributed on all chromosomes except chromosome 6H. The QTL RL_4H_D18 showed great potential by explaining 44% of the phenotypic variation of the corresponding trait, with an allele effect of 5.87. All of these QTLs are treatment-specific, i.e., adaptive QTLs, as they underlie their variation of root length under drought only. For RL_DTI, eleven QTLs were mapped: 1H (5), 3H (2), 5H (2), and 6H (2). The most powerful QTL for this trait, namely RL_5H_DTI6, accounts for 58% of the phenotypic variation. For Reduction_RL, one QTL was detected, namely, Red_5H_RL1 on 5H, which explained 38% of the phenotypic variation with a −6.2 effect for the favorable allele (Table S3).

#### 2.3.4. QTLs for SL Traits

Remarkably, no QTLs were mapped under the control or drought conditions for SL. However, two QTLs were detected for SL_DTI and Reduction_SL, namely, SL_7H_DTI1 and Red_7H_SL1. Both QTLs were mapped on chromosome 7H and explained 36% and 28% of the phenotypic variation of SL_DTI and Reduction_SL, respectively (Table S3).

#### 2.3.5. QTLs for FW Traits

Five QTLs were localized under the control condition, and four QTLs were identified under drought (Table 4). It is noteworthy that four SNP markers were found to be associated with four QTLs under the control condition as well as four QTLs under drought. These results suggested that these QTLs harbor alleles that activate under control and drought conditions, i.e., these QTLs are constitutive QTLs. The major QTLs were mapped on chromosomes 6H (FW_6H_C4 and FW_6H_D4) and explained 55% and 57% of the total variation under control and drought conditions, respectively. For FW-related indices: one QTL was mapped on chromosome 1H (FW_1H_DTI1) and one QTL on chromosome 3H for reduction (Red_3H_FW1). They explained 48% and 34% of the variation of FW_DTI and Reduction_FW, respectively (Table S3).

#### 2.3.6. QTLs for SRR Traits

In total, three QTLs were identified: two QTLs under the control condition and one QTL under drought. The SRR_1H_C1 and SRR_7H_D1 are very effective QTLs that explained 53% and 34% of the phenotypic variation in SRR under control and drought conditions, respectively.

The SNP markers that have a significant association with more than one trait are presented in Table 5. Chromosome 6H had four markers that were found to be associated with more than one trait. Three SNPs on chromosome 3H, S3_465596823 (T), S3_471113003 (C), and S3_50419767 (C), were found to be associated with increased FW under control and drought conditions. Additionally, the S7_61794629 marker on chromosome 7H was associated with increased FW under both conditions. Three markers located on chromosome 6H were associated with Reduction_G% and G%_DTI (Table S3).

The linkage disequilibrium (R^2^) between each pair of the significant SNP located on the same chromosome was calculated (Table S3). As a result, 16 highly significant LD genomic regions (R^2^ > 0.60) were detected. High LD was found among all SNPs located on chromosome 3H for GP_D. In RL_D, two separated high LD regions were found on chromosome 1H. The first region consisted of six SNPs, while the second region consisted of four SNPs. There was no high LD between the two regions.

Gene annotation was performed for each QTL found in this study (Table S3). The genomic regions flanking the physical position of the corresponding SNP marker were scanned for candidate genes in 0.5 Mbp before and after the marker. We focus on the marker that had an association with more than one trait in our discussion.

### 2.4. QTLs Associated with Drought Tolerance at Different Growth Stages

A set of 20 simple sequence repeat marker alleles that were previously reported [45], with their significant association with nine QTLs controlling drought tolerance at the adult growth stage, was tested with all traits scored in this study to find any possible causative QTLs controlling traits under different growth stages (Table S4). Out of the 9 QTLs, one QTL (DSI-GYPS-Gh) was found to be associated with Red_FW at the seedling stage (Table 6). The visible allele of this marker was considered to increase tolerance to drought by decreasing the reduction in grain yield per spike under drought conditions.

## 3. Discussion

### 3.1. Genetic Variation in Drought Tolerance at Germination and Seedling Stages

Seed germination and seedling establishment are of great importance for subsequent plant development and growth. Low water content during the seedling stage can cause a complete failure of crop production [46]. The high genetic variation found among genotypes for all traits, and high heritability estimates are very useful for barley breeding programs to efficiently select the drought-tolerant genotypes and improve drought tolerance. The genetic variation and heritability are of high importance to select for the traits of interest and to select which genotypes will do better than others under drought stress [47].

In this study, drought tolerance was evaluated based on five different traits, representing the germination and seedling stages. All five traits had a direct relation to drought stress. Recently, in a worldwide collection of barley, Thabet and Moursi et al. [7] found that all seed-germination-related and seedling-related (G%, GP, RL, and SL) traits were significantly reduced under equivalent drought stress. Similarly, the germination rate, root and shoot length, as well as seedling fresh weight, were markedly decreased under drought stress in barley [8,45,48]. It was reported that multiple-trait-based selection is more efficient than single-trait-based selection [49]. Thus, five drought tolerance indices were estimated and used to select the most drought-tolerant genotypes. As a result, a set of 13 genotypes were classified as drought-tolerant in at least two DSIs at the seedling stage. Remarkably, the same set of material was evaluated for drought tolerance at the adult growth stage in two locations [45]. PNBYT15 was among the 15 most drought-tolerant genotypes for the 1000-kernel weight (DSI-TKW-Gh and DSI-TKW-AS) and grain yield per spike (DSI-GYPS-AS and DSI-GYPS-Gh) categories. In this study, the same genotype was identified as one of the most drought-tolerant genotypes at the germination and seedling stages (G%, RL, and FW). Therefore, PNBYT15 can tolerate drought stress at different growth stages. Using PNBYT15 as a parent in future breeding programs will undoubtedly improve drought tolerance in barley. Moreover, the same set of genotypes was tested under heat stress at adult growth stages for two successive seasons [48]. Among the heat-tolerant genotypes, six genotypes—SCSAL-21, PNBYT15, SCSAL-36, SC4-41, SCSAL-52, and PNBYT27—were among the drought-tolerant genotypes in this study.

### 3.2. Phenotypic Correlation

The phenotypic correlations among traits under control conditions were higher than those under drought conditions (Figure 3). The same results were previously reported among the same traits on barley [7]. Under drought stress, there was no correlation between the germination and seedling stages. Therefore, it is very important to consider both types of traits under drought studies to select drought-tolerant genotypes. This can be partially explained by the independent distribution of QTLs for germination-related traits and those for seedling traits across the seven chromosomes of barley (Figure 5). Under drought stress and control conditions, the most significant phenotypic correlation was with RL. Root traits play an important role in tolerating drought.

As mentioned above, the same materials were tested for drought tolerance at the adult growth stage in field experiments. We tested the correlation among drought tolerance in the germination, seedling, and adult growth stages. We did not find high significant correlations among drought tolerance in the three stages, except for G%_DTI with TKW-DSI-Gh, r = 0.27 * (Table S5). These results suggest that selection for drought tolerance cannot be accomplished based on a single stage. Thus, for producing elite drought-tolerant genotypes, pyramiding the alleles associated with drought tolerance at different growth stages is necessary. Drought stress can occur in any growth stage, and genotypes performed differently through different growth stages. Similar results were obtained in wheat by Sallam and Mourad et al. [50]. Drought stress is stage- and genotype-dependent.

### 3.3. The Marker–Trait Association

In the current study, a set of 16,966 SNPs were generated from GBS and distributed on all the seven chromosomes. This number of markers allowed many QTLs for all traits under both conditions to be detected using single-marker analysis. Once SNP markers are significantly associated with the desired trait, they can be used by plant breeders for marker-assisted selection (MAS) to select individual plants that have a combination of alleles of interest from large segregated populations [51,52]. The highest number of QTLs was detected for root-related parameters (33 QTLs). This result is in accordance with earlier studies on different barley collections. Naz and Arifuzzaman et al. [53] reported that out of 28 QTLs, 19 were identified for root-related traits. Similarly, Thabet and Moursi et al. [7] found 36 QTLs for root-related parameters; Abdel-Ghani and Sharma et al. [9] mapped 34 QTLs for root-related traits under control and drought conditions. This is a further indication of the vital role of the roots under drought stress as it creates considerable differences among genotypes and allows distinction between the tolerant and susceptible groups. These results suggested that SMA is a useful approach to precisely map marker–trait associations. Moreover, the results show that these genotypes possess a sufficient level of genetic diversity to be used for MAS.

A total of 71 QTLs, distributed on all chromosomes, were detected in this study under both treatments. Highly significant LD genomic regions were found. These high genomic regions represented one QTL, and the SNPs that were in high LD tend to be coinherited together. The R^2^ of all QTLs was ranged from 0.275 to 0.582, which suggested that the detected QTLs have major effects. Interestingly, some markers were found to be associated with more than one trait. These markers had pleiotropic effects that have a very important impact on the marker-assisted selection and identification of candidate genes [54]. Hence, we will focus on those that were associated with more than one trait.

Three SNPs on the 3H chromosome were found to be associated with FW under control and drought conditions. A complete LD was found between S3_471113 and S3_50419767, indicating that these two markers are coinherited together and represented the same QTL. Both markers had nonsignificant LD with S3_471113003. Allele C of S3_471113003 had the lowest R^2^ and allele effects for FW under both conditions compared to alleles T and C for S3_471113003 and S3_50419767, respectively. These two markers, with significant LD, could be very important in a breeding program for marker-assisted selection as they are associated with increased FW under both conditions.

On chromosome 5H, the SNP marker S5_526418931 had a significant association with two QTLs, G%_5H_C6 and GP_5H_C2, indicating a pleiotropic mode of action for the genes residing in this QTL’s vicinity. For G%-related traits, the major QTL that explained 47% of the total phenotypic variation under the control condition (G%_1H_C2) was localized at 463,037,615 bp (61.83 cM). This result agreed with the results of Thabet and Moursi et al. [7], who mapped a QTL for GP_D. This result is supported by the positive and significant correlation between G% and GP under control and drought (Figure 3a,b) conditions. For GP-related parameters, it is noteworthy that all five GP_D-related QTLs were mapped on chromosome 3H, approximately at the same position of 428,008,275 bp (51.63 cM). Very close to this cluster of QTLs for GP, a cluster of QTLs for biomass and shoot dry weight was detected under drought [9]. The other SNP, located on the same chromosome, was S5_99278943, which controlled RL_DTI and Reduction_RL. There was no LD between the two SNPs due to the long physical distance between the two SNPs, suggesting that there were different QTLs on the same chromosome (Figure 5). In agreement with that, RL_DTI and Reduction_RL showed a very high negative and significant correlation, with r = −0.93 *** (Figure S1).

Three important significant SNP markers on chromosome 6H (S6_15937118, S6_15937146, and S6_520541285) revealed significant associations with QTLs for G%_DTI and Red_G%. A high LD was found between S6_15937118 and S6_15937146, while there was no significant LD between these two SNPs with S6_520541285. Presumably, these alleles regulate the variation of these traits conversely, i.e., they increase one trait and decease the second at the same time. This interpretation is supported by the significantly high negative correlation between Red_G% and G%_DTI (r = −0.93 ***). Additionally, these alleles should be very valuable for selection for drought tolerance because they orchestrate the variation under both growth conditions. Therefore, the selection for drought tolerance can be made under control conditions only or under drought. These QTLs appear to be novel as no equivalent QTLs were mapped at the same position in the earlier studies. The S6_37139810 SNP was found to be associated with FW under both treatments, and it explained 55% and 58% of the total variation for FW under control and drought conditions, respectively. The S6_37139810 SNP seemed to be more important than those that were found on chromosome 3H. Moreover, it had a higher significant association (*p*-value = 0.0002 under both conditions) with FW than the other three SNPs located on chromosome 3H.

The SNP markers that have pleiotropic effects could be converted to kompetitive allele-specific PCR (KASP) and validated in different germplasm before use for MAS.

### 3.4. Marker Validation

Sallam and Amro et al. [45] found 20 marker alleles that were associated with nine QTLs controlling grain weight and grain yield per spike under normal and drought conditions and drought susceptibility indices at the adult growth stage using SSR markers (Table S4). In the current study, we tested the association between these significant SSR markers and phenotypic data scored under both conditions. We found that one marker/QTL (stm773-2-149/DSI-GYPS-Gh) was found to be associated with the trait (Reduction- FW). This QTL (DSI-GYPS-Gh) was previously reported to be associated with increased drought tolerance by decreasing the drought susceptibility index for the trait of grain yield per spike (DSI = −0.903). Likewise, in this study, the same marker was strongly associated with the reduction in FW (*p*-value of 0.00010 and R^2^ of 22.9%). The visible allele of that marker was found to be associated with a decreased reduction in FW. This result suggested that although the different growth stages are controlled by different genes generally, detecting an interesting genomic region harboring genes controlling drought tolerance at different growth stages could be possible. Many studies have reported that fresh weight and dry weight at the seedling stage have a significant association with grain weight under various stresses. A positive and strong correlation was found between seedling FW and final grain weight in sorghum (*Sorghum bicolor* (L.) Moench) under drought stress (r = 0.89 **) [55].

### 3.5. Analysis of Gene Annotation

The candidate genes were identified for the most effective markers that had associations with more than one trait. Chromosome 6H harbored the highest number of candidate genes. The gene *HORVU6Hr1G008640* and *HORVU6Hr1G008730*, associated with G%-related traits, encode Catalse1 and Catalase3 isozymes (Table 5). It has been proven that drought is associated with high H_2_O_2_ levels. Several studies have demonstrated the pivotal role of catalase activity to equilibrate the concentration of H_2_O_2_ in the embryonic tissues during seed germination, as reviewed by Hite and Auh et al. [56]. Furthermore, on chromosome 6H, the gene *HORVU6Hr1G008880* is annotated to be heat shock 70 kDa protein C (*hsp70*). *hsp70* chaperones are needed to ensure optimal folding of the novo-synthesized proteins via preventing protein aggregation that occurs after seed rehydration. Moreover, *hsp70* ensures the rapid biosynthesis and transportation of proteins during the rapid embryogenesis that characterizes the seed germination stage [57]. The overexpression of tobacco *hsp70* enhanced drought stress [58]. The cytosolic *hsp70* transcripts dramatically increased over 50-fold under PEG-induced drought stress in barley [59]. Moreover, *HORVU6Hr1G075640* encoded the AP2-like ethylene-responsive transcription factor (AP2/ERF). AP2/ERFs positively regulated drought tolerance in *Arabidopsis thaliana* (L.) [60]. AP2/ERFs improved drought tolerance in Arabidopsis via activating the expression of Aquaporin genes [61]. AP2/ERFs negatively regulate ABA signaling during seed germination by regulating the expression of abscisic acid insensitive (ABI3, ABI4, ABI5) during seed germination and early seedling development [62]. Expression of *Ah*AP2/ERF from peanut in Arabidopsis increased ABA sensitivity and conferred drought tolerance [63]. The last two genes on chromosome 6H, *HORVU6Hr1G016350*, and *HORVU6Hr1G016260*, encode cytochrome P450 superfamily protein and transducin/WD40 repeat-like superfamily protein, respectively, and are associate with FW-related traits. The expression of the cytochrome P450 family gene, *At*CYP78A7, from Arabidopsis into rice improved drought tolerance [64]. Similarly, the overexpression of *Gm*CYP82A3 from soybean (*Glycine max* (L.) in tobacco enhanced seed germination under osmotic stress through inducing the expression of the jasmonic acid/ethylene (JA/ET) signaling pathway-related genes [65]. Supportive of that, the overexpression of wheat *Ta*WD40D positively controlled drought tolerance during seed germination and seedling development in Arabidopsis. In the same study, the suppression of *Ta*WD40D in wheat decreased seed vigor and seedling development [66]. Gachomo and Jimenez-Lopez et al. [67] found that GIGANTUS1 (GTS1), a member of the transducin/WD40 protein superfamily, regulated seed germination, growth, and biomass accumulation in Arabidopsis. Taking these findings together, the genes on chromosome 6H are potentially involved in drought tolerance in barley. Most of them regulate seed germination and biomass traits; therefore, they are potential targets to select for drought tolerance.

Notably, the genes on chromosome 3H strictly accounted for the variation of FW under control and drought conditions. Of these, the gene *HORVU3Hr1G061080* was annotated to the glutaredoxin family protein. Glutaredoxin conferred drought tolerance in maize at the seedling stage [68]. The overexpression of *Os*GRX8 from rice increased drought tolerance in Arabidopsis [69]. Under drought, *Me*GRXC15 from cassava boosted seedling development in Arabidopsis via the ABA signaling pathway; thereby, the growth parameters for transgenic plants were higher than those of wild plants [70]. Similarly, the overexpression of glutaredoxins genes LOC_Os02g40500 and LOC_Os01g27140 from rice conferred drought tolerance in Arabidopsis by inducing enzymatic antioxidants and reducing the levels of reactive oxygen species (ROS). The transgenic plants had longer roots and a higher germination percentage than the wild-type plants [71]. Additionally, *HORVU3Hr1G061120* encodes gibberellin 2-beta-dioxygenase1 (*GA2ox1*). *GAox* regulates the active gibberellin homeostasis as they are involved in biosynthesis, as well as the deactivation of gibberellin. The role of gibberellins (GAs) as a class of growth hormones in the response of plants to abiotic stress has been shown. Gibberellins positively regulate plant organ development. GA2ox1 was significantly upregulated under 10%-PEG drought stress in tea plants, indicating that GA2ox1 is involved in stress-response regulation [72]. Arabidopsis mutants overexpressing *At*GAox1 and *At*GAox2 showed lower drought tolerance [73]. These findings demonstrated that GA2ox1 negatively regulated drought tolerance via controlling the bioactive GA levels. The remaining genes on chromosome 3H encode different classes of transcription factors (TFs; Table 5). The first and the second TFs, namely, RING/U-box superfamily protein and protein kinase superfamily proteins (PKs), respectively, are involved in the positive regulation of ABA-dependent drought tolerance at seed germination and root growth in Arabidopsis [74]. Additionally, in wheat, *Ta*PK2622 can improve drought tolerance via triggering Ca2^+^ signaling and regulating the intracellular ion homeostasis [75]. The third TF is the basic helix–loop–helix (bHLH) DNA-binding family protein (bHLH). Waseem and Li [76] found that SlbHLH22, when overexpressed, confers higher seedling vigor and drought tolerance in transgenic tomato plants relative to wild-type plants. That effect was caused by high activation in the enzymatic antioxidant machinery components as well as antioxidant-related genes. The fourth TF is in the late embryogenesis abundant (LEA) hydroxyproline-rich glycoprotein family. In *Brassica napus*, four LEA transcription factors were found to be negative regulators of GA signaling. LEA proteins conferred drought tolerance through their involvement in the ABA signaling pathway [77]. Given these findings, it seems that GA and LEA are working antagonistically; GA increases organ development, and, on the contrary, LEA diminishes GA levels and, consequently, reduces organ development. This interdependency may help the plant to channel resources to withstand drought rather than for organ development. Overall, genes on chromosome 3H are diverse, including regulatory genes such as TF-coding genes and functional genes such as antioxidant-related genes. This result suggests that barley combines different mechanisms to cope with drought. Moreover, most of these genes are involved in the ABA signaling pathway, suggesting a compensatory mode of action. Of high importance is that all genes on chromosome 3H are attributed to the variation of FW under control and drought conditions; thus, 3H represents a target to select for FW improvement.

Three genes on chromosome 5H control the variation of root length and germination parameters. Of these, two genes encode ATP-binding cassette (ABC) transporter G family member 5 and ABC transporter G family member 1 (Table 5). Both genes account for the variation of RL-related traits. The ABC transporters are involved in ABA transport under drought stress. Arabidopsis *At*ABCG25 transporter was greatly expressed in vascular tissues; it is an ABA efflux carrier [78]. In the current study, ABC transporters are involved in root length variation. Supportive of our results, the ABC transporter is mainly expressed in several organs, including leaves and roots [78]. Kim and Jin et al. [79] reported that the overexpression of *At*ABCG36 enhanced drought tolerance that was demonstrated by a 1.7-fold increase in shoot biomass and a 1.2-fold increase in root length. Upon prolonged water deficit, ABC transporters were responsible for translocating root-derived ABA via the xylem to the aboveground plant parts [80]. Altogether, ABC transporters are responsible for ABA transportation under drought, and they accounted for the root response. Our conclusion is supported by previous research [81] that reported that another member of the ABC transporters, *At*ABCG16, peaked in the root plasma membrane. The silencing of *St*ABCG1 in *Solanum tuberosum* L., which is mainly expressed in the roots, resulted in alterations in root and tuber morphology as well as hypersensitivity to drought [82]. A further gene on chromosome 5H encodes *At*4g40080-like protein, which accounts for variation in G% and GP (Table 5). This protein has clathrin-dependent endocytosis in the Golgi apparatus and includes the epsin N-terminal homology (ENTH) domain. Clathrin-mediated endocytosis was involved in the seed germination of *Gastrodia elata* [83]. Endocytosis is triggered immediately upon seed imbibition, but Arabidopsis seed germination was greatly delayed when endocytosis was inhibited under drought [84]. This result suggested that endocytosis has a pivotal role in seed germination and root development.

Two genes on chromosome 7H account for SL-related trait variations (Table 5), which encode dehydrogenase/reductase SDR family member 4 (DHRS4) and glutathione peroxidase 1 (GPX1). DHRS4 was upregulated under drought stress in grapevine, indicating its involvement in the drought response [85]. This gene has an oxidoreductase activity, with a prominent role in shoot apex development and shoot apical meristem maintenance in Arabidopsis [86]. The second gene encodes GPX1. Arabidopsis GPX1 transcripts were found to be abundant in shoots of wild-type plants as well as mutant plants [87]. GPX is the main constituent of the ascorbate–glutathione (ASC–GSH) cycle that is the main cycle for detoxifying excess H_2_O_2_ produced upon drought [88]. Under drought, the induction of AsA–GSH-related genes significantly increased the drought tolerance in wheat seedlings, as demonstrated by increased shoot lengths and shoot dry weights, along with a low H_2_O_2_ content [89].

## 4. Materials and Methods

### 4.1. Plant Material

The diverse set consisted of 60 spring barley genotypes. The biological status, pedigree information, and population structure have been illustrated [90]. The list of all genotypes is presented in Table S1.

#### Seed Germination Test

All genotypes were tested under two treatments: control (0-PEG) and 20%-PEG for drought stress. For control (0-PEG) as well as for drought stress (20%-PEG), each genotype was replicated three times in a randomized complete block design (RCBD). In each replicate, 20 seeds from each genotype were spread in a 9-cm diameter Petri dish on 2 filter papers, Whatman No.1, moistened by 10 mL of the corresponding solution; the Petri dishes were incubated under 20 °C in the darkness. The seed germination was scored every 24 h for up to ten days. The seed was considered germinated when the radicle length was 2 cm. Twenty-two seed germination and seedling traits were scored, as described in Table 1. Germination parameters were assessed according to International Seed Testing Association rules (ISTA); Table 1 summarizes the names, abbreviations, and estimation descriptions for all traits.

For shoot length and root length measurements, 10 seeds from each genotype were placed in a piece of rolling paper [91]; the papers were placed in 1 L beakers approximately filled with water for the control treatment or 20%-PEG for the drought treatment. The 20%-PEG solution was replaced every second day until the end of the experiment. After 12 days, the shoot length (SL) and root length (RL; in cm) were manually measured. The fresh weight (FW) was recorded (g) using a sensitive balance (Sartorius AC 1215, Germany). The shoot- and root-related parameters—shoot length/root length ratio (SRR), reduction of shoot length (Reduction_SL), reduction of root length (Reduction_RL), drought tolerance index of shoot length (DTI-SL), and drought tolerance index of root length—were calculated as described in Table 1.

### 4.2. Data Analyses

Analysis of variance (ANOVA), broad-sense heritability (H^2^), and correlation coefficients were calculated using PLABSTAT [92] and R software.

Principal component analysis (PCA) under control and drought conditions was calculated for all traits. PCA converts the values of the potentially related traits into an uncorrelated measure called principal components. The PCA dimensions are arranged in a descending manner, where the first dimension encompasses the highest observed variance, giving the best-estimated variance in the experiment. The PCA analysis revealed that RL, SL, and FW attributed to the most variation in this collection. The PCA was computed in accordance with Julkowska et al. [93].

### 4.3. DNA Extraction and Genotyping-by-Sequencing (GBS)

Four-to-five leaves from each genotype (five-day-old plants) were collected to extract DNA. The extraction protocol was DNAzol reagent (Molecular Research Center, Inc. Technical Bulletin 6). The concentration of DNA for each genotype was measured using spectrophotometry (Gen5TM microplate reader and image software, with Take3TM microvolume plates from BioTek, Winooski, VT, United States) and prepared for GBS. The DNA was genotyped using GBS by digesting the DNA with two different restriction enzymes, *PstI,* and *MspI*, as described in Poland and Rife [94]. Pooled libraries were sequenced using Illumina Inc. NGS platforms. The reads of the sequence were used for single nucleotide polymorphism (SNP) calling using a TASSEL 5.0 v2 GBS pipeline [95]. Barley cv. Morex (version MorexV2, as a reference genome) was used for identifying SNP markers, their physical position, and localization. The SNP markers identified were filtered for minor allele frequency (MAF > 0.05), maximum missing sites per SNP < 20%, and maximum missing sites per genotype < 20%. Heterozygous loci were then marked as missing to obtain better estimates of marker effects (Peter Bradbury, personal communication).

Single-marker analysis (SMA) was used to test the associations between SNP markers, and all phenotypic data scored on all genotypes. The analysis was done using R software following this model:

$$Y\;=\;µ\;+\;f\;\left(\mathrm{marker}\right)\;+\;\mathrm{error},$$

where Y is equal to the value of the respective trait value, μ refers to the mean of the population, and f (marker) is a function of the significant markers [96].

### 4.4. Candidate Gene Identification

The physical positions of the significant SNPs were used to find the candidate genes, which colocalize or are very close to them (around 0.5 Mbp). We used a recent barley genome dataset and geneset (BARLEX; http://apex.ipk-gatersleben.de) to annotate the genes as candidates. The physical positions in base pairs and their corresponding genetic position in centiMorgan (cM) of the significant SNPs were detected using the most recent maps of barley [97,98].

### 4.5. QTLs Controlling Drought Tolerance at Different Growth Stages

A set of 20 SSR marker alleles controlling nine QTLs, reported by Sallam et al. [45], was tested in this study, with all traits scored at the germination and seedling stages. The QTL names, traits, and significant SSR markers are listed in Table S4. The association was examined using single-marker analysis, as described above.

## 5. Conclusions

Taking these results together, this diverse barley collection contained adequate phenotypic diversity to better understand drought stress on germination and early seedling growth. Interestingly, this material was evaluated for drought tolerance at three important growth stages, and we concluded that the PNBYT15 genotypes can tolerate drought in all these three growth stages. This conclusion was shown by the high consistency with other studies, as several clusters of QTLs for various traits were detected in nearly the same positions. Both the drought-tolerant genotypes and the sensitive ones are of high importance. The former can be used further in breeding programs as donors of the favorable alleles, while the sensitive ones can be used as contrasting parents to develop new mapping populations to better understand drought stress tolerance. The effective alleles are valuable to select for improving several traits at once under drought stress. The drought tolerance index is a valuable criterion for selection. The analysis of gene annotation confirmed the power of SMA to detect the association between SNPs and target traits. The consistency of our results with previous studies that have harnessed larger populations and different marker–trait association methods suggests that single-marker analysis is successful in identifying significant comparable associations. The identified candidate genes are of high importance to select for improving numerous traits; genes on chromosomes 3H, 5H, 6H, and 7H are potential genes to select for FW, RL, G%, and SL, respectively. Finally, the marker stm773-2-149, which was found to be associated with drought tolerance at germination, seedling, and adult growth stages, is very important and can be used for marker-assisted selection.

## Figures and Tables

**Table 1:** The names, abbreviations, and descriptions of measurement for all estimated traits in 60 types of Egyptian spring barley.

Name	Abbreviation	Description of Measurement
Germination Percentage	G%	$G\%=\frac{n}{N}$×100, where n is the number of germinated seeds at the end of the experiment, N is the total number of seeds.
Germination Pace	GP	$\mathrm{GP}=\frac{N}{\sum \left(n\;\times \;g\right)}\times 100,$ where N is the number of germinated seeds at the end of the experiment, n is the number of newly germinated seed at certain day g, g = (1, 2, 3, …)
Root Length	RL	Root length was measured with a scaled ruler (in cm)
Shoot length	SL	Shoot length was measured with a scaled ruler (in cm)
Shoot–Root Length Ration	SRR	as the ratio of the SL to the RL
Fresh Weight	FW	Fresh weight was recorded in grams using a sensitive balance (Sartorius AC 1215, Germany)
Reduction of Germination Percentage	Reduction_G%	Reduction of G% = G% under control − G% under drought
Reduction of Germination Pace	Reduction_GP	Reduction of GP = GP under control − GP under drought
Reduction of Root Length	Reduction_RL	Reduction of RL = RL under control − RL under drought
Reduction of Shoot Length	Reduction_SL	Reduction of SL = SL under control − SL under drought
Reduction of Fresh Weight	Reduction_FW	Reduction of FW = FW under control − FW under drought
Drought Tolerance Index (Germination Percentage)	G%_DTI	$G\%\_\mathrm{DTI}=\frac{G\%\;\mathrm{under}\;\mathrm{drought}}{G\%\;\mathrm{under}\;\mathrm{control}}\;\times \;100$
Drought Tolerance Index (Germination Pace)	GP_DTI	$\mathrm{GP}\_\mathrm{DTI}=\frac{\mathrm{GP}\;\mathrm{under}\;\mathrm{drought}}{\mathrm{GP}\;\mathrm{under}\;\mathrm{control}}\;\times \;100$
Drought Tolerance Index (Root Length)	RL_DTI	$\mathrm{RL}\_\mathrm{DTI}=\frac{\mathrm{RL}\;\mathrm{under}\;\mathrm{drought}}{\mathrm{RL}\;\mathrm{under}\;\mathrm{control}}\;\times \;100$
Drought Tolerance Index (Shoot Length)	SL_DTI	$\mathrm{SL}\_\mathrm{DTI}=\frac{\mathrm{SL}\;\mathrm{under}\;\mathrm{drought}}{\mathrm{SL}\;\mathrm{under}\;\mathrm{control}}\;\times \;100$
Drought Tolerance Index (Fresh Weight)	FW_ DTI	$\mathrm{FW}\_\mathrm{DTI}=\frac{\mathrm{FW}\;\mathrm{under}\;\mathrm{drought}}{\mathrm{FW}\;\mathrm{under}\;\mathrm{control}}\;x\;100$

**Table 2:** Analysis of variation for the traits scored on 60 types of Egyptian spring barley under control (0-PEG) and drought (20%-PEG) conditions.

Source of Variance	Germination Percentage	Germination Pace	Shoot Length	Root Length	Shoot/Root Ration	Fresh Weight
Treatments (T)	118.77 **	115.76 **	591.64 **	467.03 **	333.09 **	29.58 **
Replications	1.76	5.64 **	0.36	0.01	3.29 *	0.03
Genotypes (G)	14.55 **	9.19 **	23.92 **	43.05 **	57.57 **	26.83 **
T × G	10.83 **	5.19 **	15.57 **	20.46 **	12.30 **	12.60 **

*, ** significant at *p* ≤ 0.05 and *p* ≤ 0.01, respectively.

**Table 3:** List of the most drought-tolerant genotypes based on the five drought tolerance indices for the corresponding traits.

Genotype	Traits	G%	GP	SL	RL	FW
SCSAL-21	3	×	×			×
PNBYT15	3	×			×	×
SCSAL-36	3	×			×	×
PNBYT1	3	×			×	×
SC4-41	2		×	×		
SCBNB57	2	×				×
SCSAL-52	2		×		×	
SCYT-28	2		×		×	
PNBYT27	2	×		×		
SC2-19	2	×				×
INTROD-46	2	×			×	
SCSAL-10	2	×				×
Giza135	2	×		×		

**Table 4:** Number of quantitative trait loci (QTLs) for seed germination and seedling establishment related traits per chromosome and under each treatment in 60 types of Egyptian spring barley under control (0-PEG) and drought (20%-PEG) conditions.

	Treatment	No. of QTL	Chromosome.	R^2^
**Traits**				
Root Length Drought	C	-	-	-
	D	21	1, 2,3,4, 7	26.9–44.3%
Shoot Length/Root Length Ratio Control	C	1	7	34.3%
	D	2	1	52.7%
Germination Percentage	C	7	1, 2, 5	29.1–47.0%
	D	3	1, 2, 6	32.1–42.9%
Germination Pace	C	2	2, 5	38.8–46.1%
	D	5	3	35.7–43.9%
Fresh Weight	C	4	3, 6	27.5–55.1%
	D	4	3, 6	28.7–57.5%
QTL under Control	15			
QTL under Drought	35			
Total	50			
**Drought tolerance index**				
Fresh Weight Drought Tolerance Index		1	1	34.2%
Shoot Length Drought Tolerance Index		1	7	36.2%
Drought Tolerance Index		3	6	30.9–33.1%
Shoot Length Drought Tolerance Index		11	1, 2, 7	28.0–58.2%
Total	16			
**Reduction index**				
Reduction of Fresh Weight		1	3	47.6%
Reduction of Shoot Length		1	7	28.3%
Reduction of Root Length		1	5	37.8%
Shoot Length/Root Length Ratio Drought		1		
Fresh Weight Control		1	2	30.2%
Total	5			
Total Number of QTLs for all traits	71			

C stands for control, D stands for drought, and R^2^ phenotypic variation explained by marker alleles.

**Table 5:** Candidate genes associated with the most effective markers and the annotation of genes.

Marker	Chr	SNP Position	Traits	Candidate Genes	Annotation
S3_465596823	3H	465596823	FW_C FW_D	HORVU3Hr1G061080 HORVU3Hr1G061120	Glutaredoxin family protein Gibberellin 2-beta-dioxygenase 1
S3_471113003	3H	471113003	FW_C FW_D	HORVU3Hr1G061850 HORVU3Hr1G061860	RING/U-box superfamily protein Protein kinase superfamily protein
S3_50419767	3H	50419767	FW_C FW_D	HORVU3Hr1G018980 HORVU3Hr1G019070	Basic helix-loop-helix (bHLH) DNA-binding family protein Late embryogenesis abundant (LEA) hydroxyproline-rich glycoprotein family
S5_99278943	5H	99278943	RL_DTI Reduction_RL	HORVU5Hr1G021110 HORVU5Hr1G021120	ABC transporter G family member 5 ABC transporter G family member 1
S5_526418931	5H	526418931	G%_C GP_C	HORVU5Hr1G069950	At4g40080-like protein
S6_15937118	6H	15937118	Reduction_G% G%_DTI	HORVU6Hr1G008640 HORVU6Hr1G008520	Catalase 1 Chromosome 3B genomic scaffold
S6_15937146	6H	15937146	Reduction_G% G%_DTI	HORVU6Hr1G008730 HORVU6Hr1G008880	Catalase 3 Heat shock 70 kDa protein C
S6_37139810	6H	37139810	FW_C FW_D	HORVU6Hr1G016350 HORVU6Hr1G016260	Cytochrome P450 superfamily protein Transducin/WD40 repeat-like superfamily protein
S6_520541285	6H	520541285	Reduction_G% G%_DTI G%_D	HORVU6Hr1G075640	AP2-like ethylene-responsive transcription factor
S7_61794629	7H	61794629	SL_DTI Reduction_SL	HORVU7Hr1G030700 HORVU7Hr1G030810	Dehydrogenase/reductase SDR family member 4 Glutathione peroxidase 1

**Table 6:** Previously reported QTL associated with drought tolerance at the adult growth stage.

QTL Name	Marker Allele	*p-*Value	R^2^	Allele Eff ^1^	Traits in This Study	Traits in Earlier Studies
DSI-GYPS-Gh	stm773-2_149	0.00010	22.9	−0.433	Red_FW	Drought susceptibility index for grain yield per spike (Sallam et al. 2019)

^1^ The effect of the visible allele.

## Supplementary Materials

The following are available online at https://www.mdpi.com/2223-7747/9/11/1425/s1. Figure S1: Correlations of the drought tolerance indices in 60 Egyptian spring barley. G%_DTI = germination percentage drought tolerance index, GP_DTI = germination pace drought tolerance index, SL_DTI = shoot length drought tolerance index, RL_DTI = root length drought tolerance index, FW_DTI = fresh weight drought tolerance index, Reduction_G% = reduction of germination percentage, Reduction_GP = reduction of germination pace, Reduction_SL = reduction of shoot length, Reduction_RL = reduction of root length, and Reduction_FW = reduction of fresh weight. X indicates the nonsignificant correlations at p = 0.05. Table S1: The biological status and pedigree information of the collection of 60 types of Egyptian spring barley. Table S2: Ranges, means, and heritability for all traits estimated in 60 Egyptian barley genotypes under control and drought stress conditions during seed germination and seedling establishment. Table S3: Detailed description of the single-marker analysis, linkage disequilibrium, and gene annotation, including QTLs and the candidate genes residing in the intervals among these linked markers. Table S4: List of QTLs associated with drought tolerance at the adult growth stage found by Sallam et al. [45]; all these markers were tested in the current study; the marker associated with traits in the current study as well as with traits at the adult stage is highlighted yellow. Table S5: Phenotypic correlation analysis among drought tolerance genes at different growth stages (germination, seedling, and adult growth stages). Table S6: Raw data for the selection indices of the respective traits under drought stress at seed germination, seedling development, and adult stages of spring barley.

## Abbreviations

PEG-6000 = polyethylene glycol 6000, ANOVA = analysis of variance, SMA = single-marker analysis, SNP = single nucleotide polymorphism, R^2^ = the ratio of phenotypic variation explained by a QTL, C = control, D = drought, H^2^ = heritability, PCA = principal component analysis, GBS = genotyping by sequencing, and r^2^ = linkage disequilibrium (LD). Traits’ nomenclature, abbreviations, and description: Germination Percentage (G%) = germinated seeds/total number of seeds × 100; Germination Pace (GP) = GP = N/∑ (*n* × g) × 100, where N is the number of germinated seeds at the end of the experiment, *n* is the number of newly germinated seeds at a certain day g, g = (1, 2, 3, …); Shoot length (SL) = the shoot length measured by a regular ruler (cm); Root length (RL) = the root length measured by a regular ruler (cm); Shoot/Root length ration (SRR) = Shoot length/Root length; Fresh weight (FW) = fresh weight recorded in grams using a sensitive balance (Sartorius AC 1215, Germany); Reduction of Germination Percentage (Reduction_G%) = G% control − G% drought; Reduction of Germination Pace (Reduction_GP) = GP control − GP drought; Reduction of Shoot length (Reduction_SL) = SL control − SL drought; Reduction of Root length (Reduction_RL) = RL control − RL drought; Reduction of Fresh weight (Reduction_FW) = FW control − FW drought; Drought Tolerance Index of G% (G%_DTI) = G% control/G% drought × 100; Drought Tolerance Index of GP (GP_DTI) = GP control/GP drought × 100; Drought Tolerance Index of SL (SL_DTI) = SL control/SL drought × 100; Drought Tolerance Index of RL (RL_DTI) = RL control/RL drought × 100; Drought Tolerance Index of FW (FW_DTI) = FW control/FW drought × 100; Traits’ names consist of the abbreviation of the respective trait, followed by a symbol for the treatment (for example, G%_C = Germination percentage control); QTL names consist of the abbreviations of trait, chromosome, and treatment, followed by a number (for example, RL_1H_D1 = QTL number one for root length on chromosome 1H under drought stress treatment).
